# Supplementary material for: Role of MalQ Enzyme in a Reconstructed Maltose/Maltodextrin Pathway in Actinoplanes sp. SE50/110
Source: Microorganisms. 2024 Jun 18;12(6):1221. doi: 10.3390/microorganisms12061221 (PMC11205506; doi:10.3390/microorganisms12061221)
Supplement: Supplementary file 1 [file microorganisms-12-01221-s001.zip › microorganisms-3056185-supplementary.pdf]

microorganisms

Supplementary materials: Role of MalQ protein in a reconstructed maltose/maltodextrin pathway in *Actinoplanes* sp. SE50/110

Camilla März, Sophia Nölting, Lars Wollenschläger, Alfred Pühler and Jörn Kalinowski

Table S1. Oligonucleotides used in this study.

Name	Sequence (5′ → 3′)
*malQ*_fw	GCATCATCATCATCATCATGGATCCGACGCGCGACTCGAGGC
*malQ*_rv	CCGCCAAAACAGCCAAGCTTCATGGCGTGGTCCCTTCCGTC
*amlE*_fw	GCATCATCATCATCATCATGGATCCACCGACCAACTGATCGCACC
*amlE*_rv	CTCATCCGCCAAAACAGCCATTAGACGGACCACCACACGG
*malZ*_fw	GCATCATCATCATCATCATGGATCCAACGACTGGGTCGAGCACG
*malZ*_rv	CCGCCAAAACAGCCAAGCTTCAGCCCCGCAGGATCGACCAGCC
*malP*_fw	GCATCATCATCATCATCATGGATCCGATCTTCGCCAAGGCAGCCAG
*malP*_rv	CCGCCAAAACAGCCAAGCTTTAATGGGGAACCGGAACCGG

Table S2. Plasmids used in this study.

Plasmid	Description	Source/Reference
pJOE5751.1	pBR322-based L-rhamnose-inducible vector, His_6-eGFP, Amp^R	Hoffmann et al. 2012
pJOE5751.1-*7587*	pJOE5751.1 containing *malQ*^{As} (ACSP50_7587) gene	This study

pJOE5751.1-*4430*	pJOE5751.1 containing *malZ*^{As} (ACSP50_4430) gene	This study
pJOE5751.1-*2474*	pJOE5751.1 containing *amlE* (ACSP50_2474) gene	This study
pJOE5751.1-*6911*	pJOE5751.1 containing *malP*^{As} *(ACSP50_6911)* gene	This study
pJOE5751.1-*acbK*	pJOE5751.1 containing *acbK* *(ACSP50_3602)* gene	Nölting et al., 2023

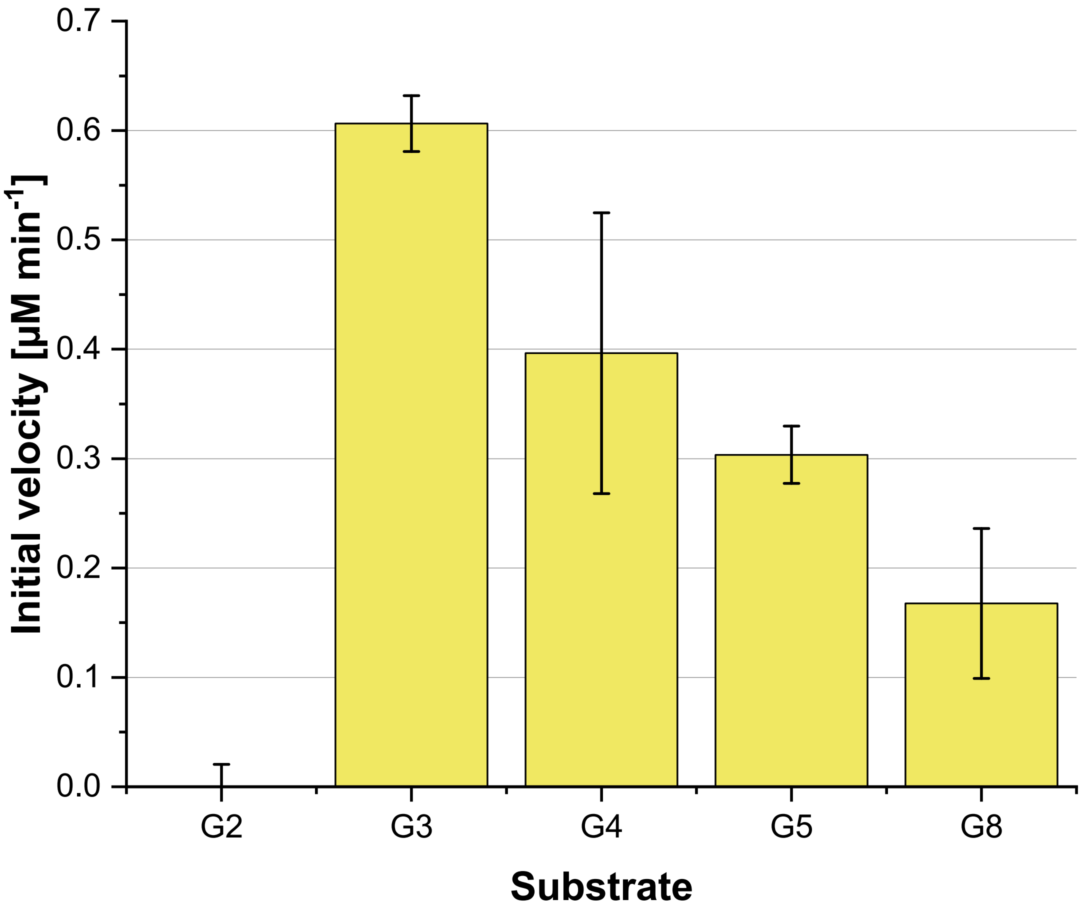

Figure S1. Characterization of 4 µM purified MalZ^{As} substrate spectrum. NADPH release from a hydrolytic enzyme assay was measured spectrophotometrically by absorption at 340 nm. Initial velocities were calculated from a standard curve. Used substrates were

maltose (G2), maltotriose (G3), maltotetraose (G4), maltopentaose (G5), and maltooctaose (G8). Negative control reaction was performed with heat inactivated MalZ^{As}. All measurements were performed in triplicate (n = 3). Standard deviations are given.

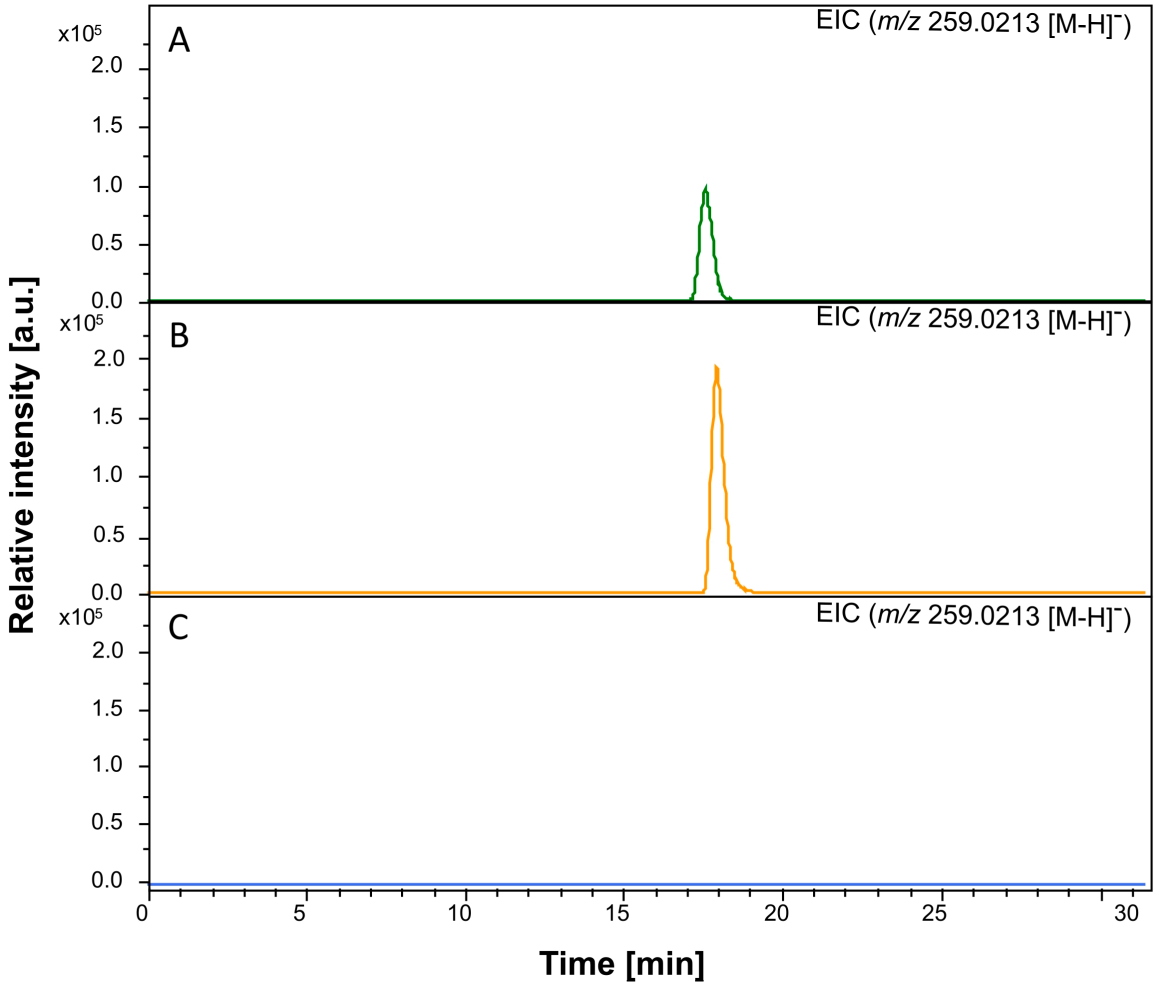

Figure S2. LC-ESI-MS analysis of MalP^{As} reaction mixture with maltopentaose as substrate after 5 hours incubation. ESI (-) EIC for glucose-1P m/z 259.0213 [M-H]$^-$. **A.** Glucose 1-phosphate as standard control, **B.** MalP^{As} assay with maltopentaose as substrate, C. Negative control with inactivated enzyme.

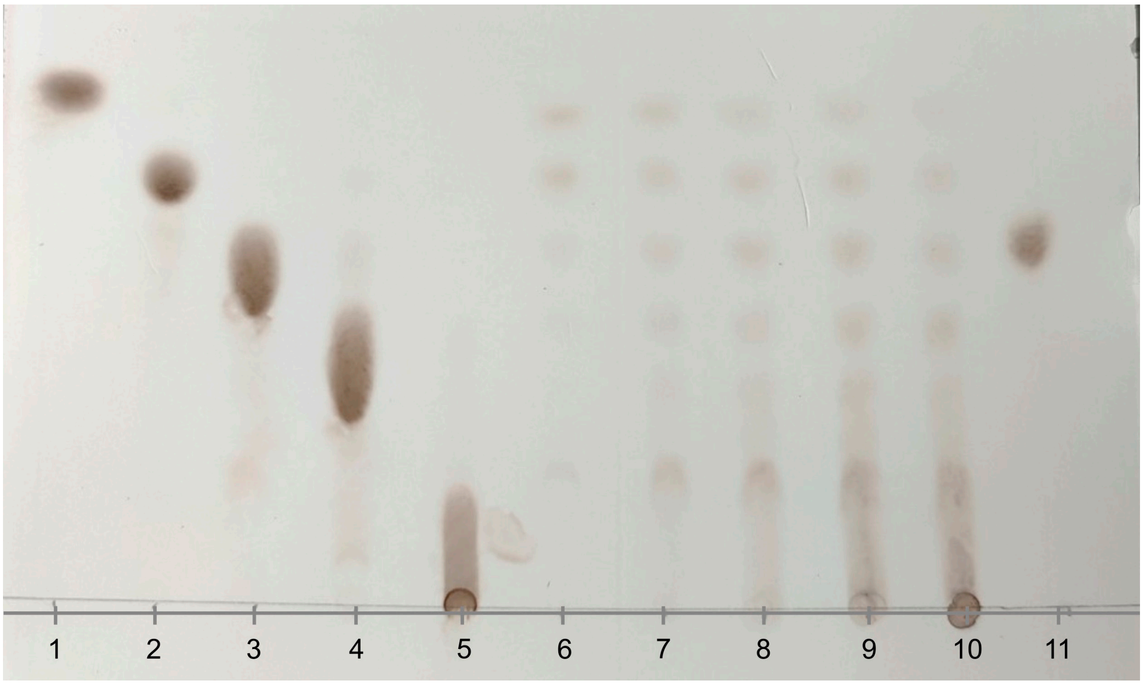

Figure S3. TLC analysis of enzymatic function of MalQ^{As} in assays in combination with various substrates. Lane 1: standard sugar glucose, lane 2: standard sugar maltose, lane 3: standard sugar maltotriose, lane 4: standard sugar maltotetraose, lane 5: standard sugar maltooctaose, lane 6: MalQ^{As} assay with maltose, lane 7: MalQ^{As} assay with maltotriose, lane 8: MalQ^{As} assay with maltotetraose, lane 9: MalQ^{As} assay with maltopentaose, lane 10: MalQ^{As} assay with maltooctaose, lane 11: heat inactivated MalQ^{As} with maltotriose.

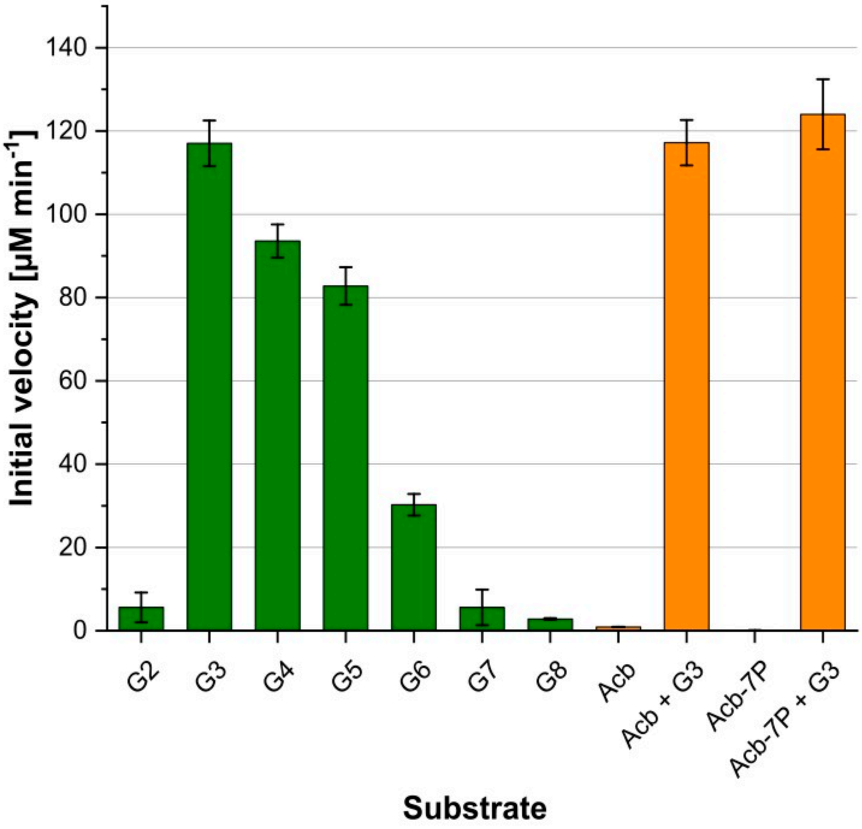

Figure S4. Characterization of purified MalQ^{As} with different substrates to evaluate the substrate spectrum. The NADPH release from a hydrolytic enzyme assay was measured spectrophotometrically by absorption at 340 nm. Initial velocities were calculated from a standard curve. Used substrates were linear α-1,4-glucans (maltose (G2), maltotriose (G3), maltotetraose (G4), maltopentaose

(G5), maltoheptaose (G7), and maltooctaose (G8)) and acarviosyl metabolites as only substrate and in combination with maltotriose (acarbose (Acb), acarbose 7-phosphate (Acb-7P)). Negative control reaction was performed with inactivated MalQ^{As} (n = 3).

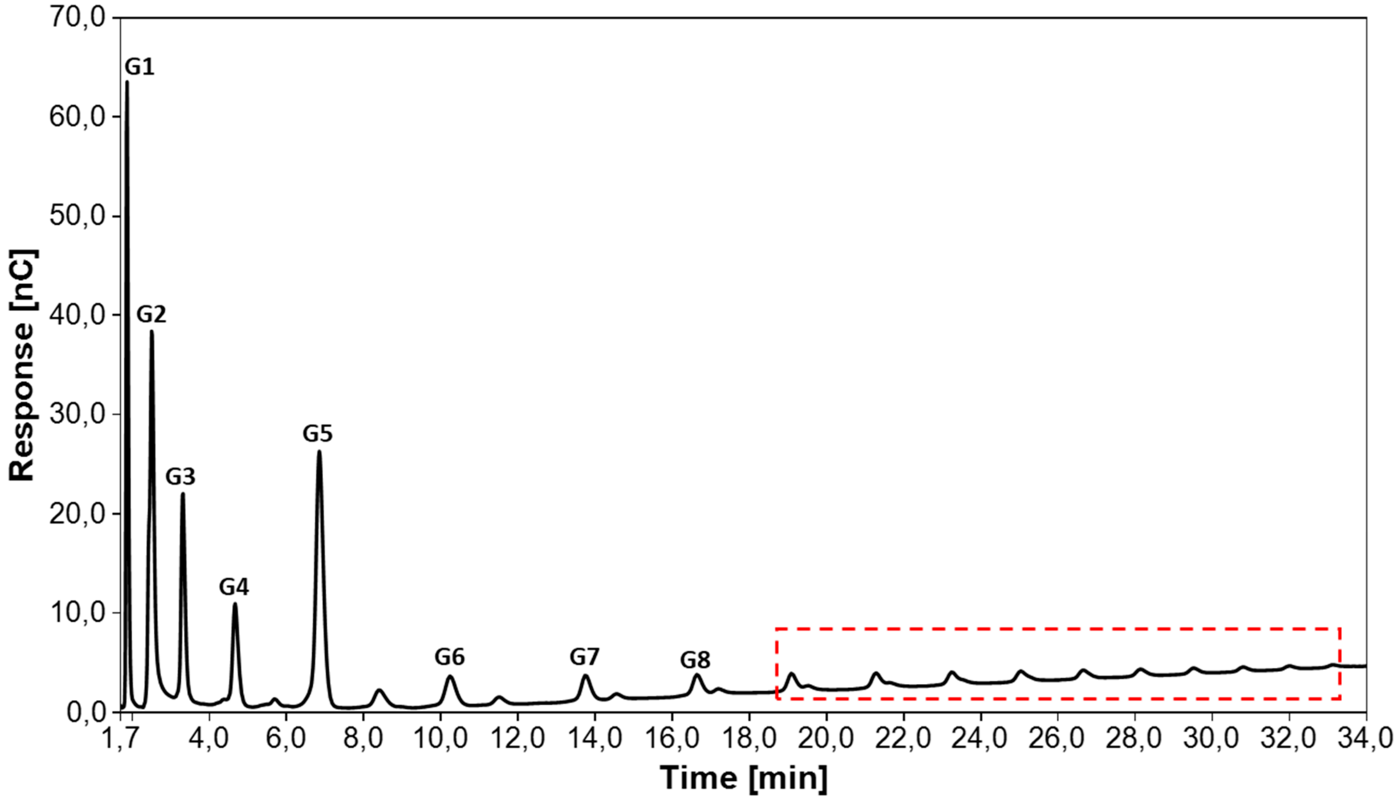

Figure S5. HPAEC-PAD chromatograms of in vitro MalQ^{As} assay with maltopentaose as substrate after 5 hours of incubation. Detection of α-1,4-glucans with different chain lengths (G1–G8). A 400-fold dilution of enzyme reaction shows multiple new peaks with longer retention time than the longest substrate maltooctaose (red box).

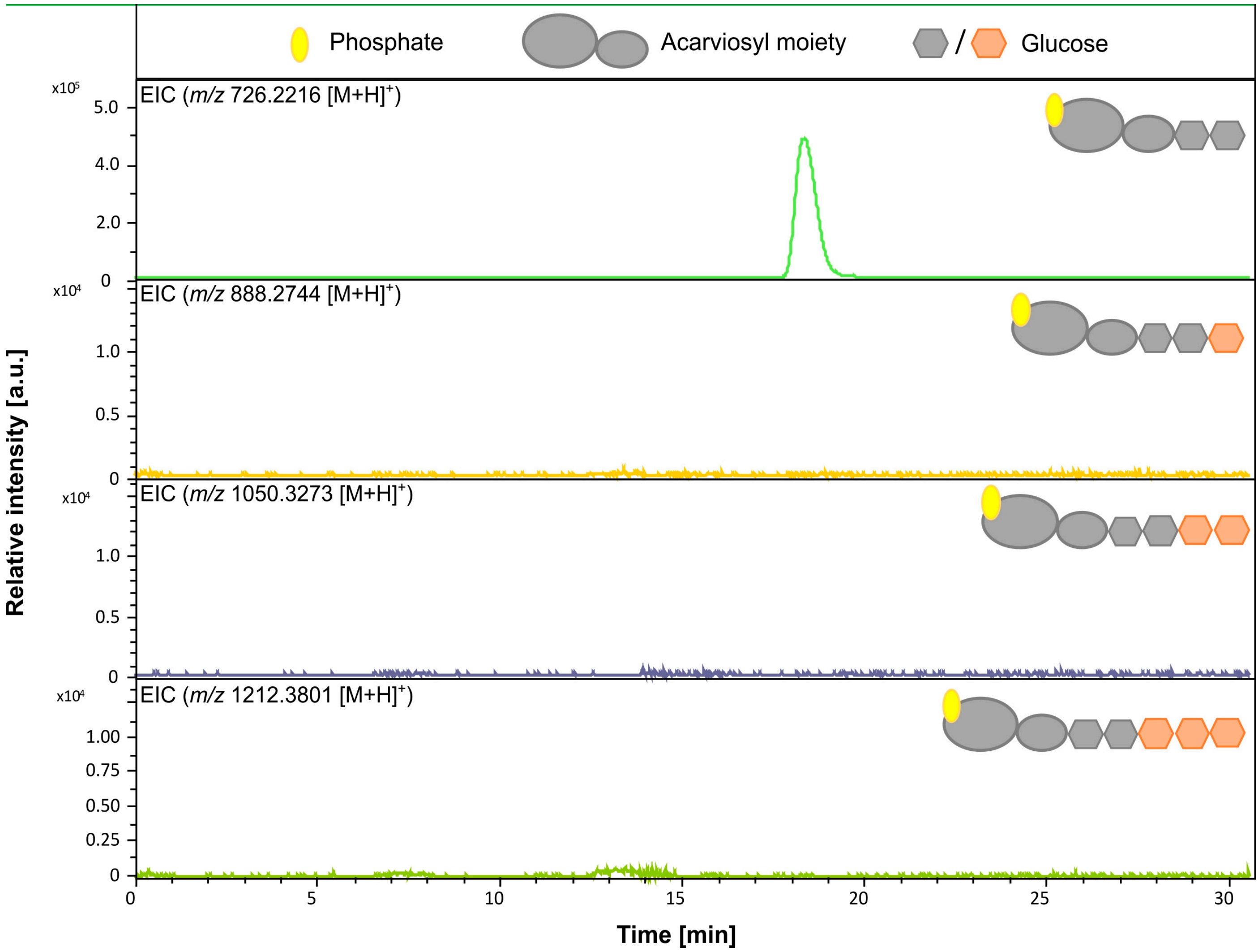

Figure S6. LC-ESI-MS analysis of MalQ^{As} reaction products. In vitro reaction mixture was incubated with acarbose 7-phosphate and maltotriose as substrates. No reaction products were identified. ESI (+) EIC for acarbose 7-phosphate m/z 726.2216, ESI (+) EIC for acarviosyl-maltotriose 7-phosphate m/z 888.2744, ESI (+) EIC for acarviosyl-maltotetraose 7-phosphate m/z 1050.3273, and ESI (+) EIC for acarviosyl-maltopentaose 7-phosphate m/z 1312.3801.

Table S3. Overview of acarviosyl metabolites originating from acarbose 7-phosphate. Mass list of phosphorylated acarviosyl metabolites including their sum formula, monoisotopic neutral mass, and $[M + H]^+$.

**Acarviosyl Metabolites**	**Abbreviation**	**Sum Formula**	**Monoisotopic Neutral Mass $[M^+]$ *m/z***	**$[M + H]^+$ *m/z***
Acarviosyl-maltose 7-phosphate (Acarbose 7-phosphate)	Ac-G2-7P	$C_{25}H_{44}NO_{21}P$	725.2143	726.2216
Acarviosyl-maltotriose 7-phosphate	Ac-G3-7P	$C_{31}H_{54}NO_{26}P$	887.2672	888.2744
Acarviosyl-maltotetraose 7-phosphate	Ac-G4-7P	$C_{37}H_{64}NO_{31}P$	1049.3200	1050.3273
Acarviosyl-maltopentaose 7-phosphate	Ac-G5-7P	$C_{43}H_{74}NO_{36}P$	1211.3728	1212.3801

Acarviosyl-maltohexaose 7-phosphate	Ac-G6-7P	$C_{49}H_{84}NO_{41}P$	1373.4256	1374.4329
